# A distinct tau oligomer strain defines the molecular and proteomic landscape of rapidly progressive Alzheimer’s disease

**DOI:** 10.1007/s00401-026-02998-4

**Published:** 2026-03-18

**Authors:** Tayyaba Saleem, Wiebke Möbius, Matthias Schmitz, Angela da Silva Correia, Carolina Thomas, Sezgi Canaslan, Peter Hermann, Stefan Göbel, Saima Zafar, Elisabeth Root, Christine Stadelmann, Olivier Andreoletti, Michael Hoppert, Tiago Fleming Outeiro, Isidre Ferrer, Neelam Younas, Inga Zerr

**Affiliations:** 1 Department of Neurology, University Medical Center Göttingen, Robert-Koch-Straße 40, 37075 Göttingen, Germany; 2 German Center for Neurodegenerative Diseases (DZNE), Von-Siebold-Straße 3A, 37075 Göttingen, Germany; 3 Department of Neurogenetics, Electron Microscopy City Campus, Max Planck Institute for Multidisciplinary Sciences, Göttingen, Germany; 4 Department of Neuropathology, University Medical Center, Göttingen, Germany; 5 Centre for Neuropathology and Brain Research, Paul-Flechsig Institute, Leipzig, Germany; 6 Biomedical Engineering and Sciences Department, School of Mechanical and Manufacturing Engineering (SMME), National University of Sciences and Technology (NUST), Islamabad, Pakistan; 7 UMR INRA ENVT 1225-Interactions Hôte Agent Pathogène–École Nationale Vétérinaire de Toulouse, Toulouse, France; 8 Institute of Microbiology and Genetics, Georg-August-University Göttingen, Grisebachstr. 8, 37077 Göttingen, Germany; 9 Experimental Neurodegeneration, University Medical Center Göttingen, Waldweg 33, 37073 Göttingen, Germany; 10 Translational and Clinical Research Institute, Faculty of Medical Sciences, Newcastle University, Framlington Place, Newcastle upon Tyne, NE2 4HH UK; 11 Emeritus Professor, University of Barcelona, Barcelona, Spain

**Keywords:** Alzheimer’s disease, Tau oligomers, Proteomics, Rapidly progressive Alzheimer’s disease, Mitochondrial dysfunction, Protein aggregation

## Abstract

Rapidly progressive Alzheimer’s disease (rpAD) is a rare subtype with rapid decline, but its molecular underpinnings remain poorly defined. Here, brain-derived tau oligomers (TauO) were systematically compared across nondemented controls, slowly progressive AD (spAD), and rpAD to test whether subtype-specific TauO signatures align with clinical aggressiveness. TauO were immunoprecipitated from frontal cortex using T22 antibody and characterized by Western blotting, transmission electron microscopy, label-free quantitative proteomics, and SH-SY5Y toxicity assays, complemented by longitudinal analysis of tau phosphorylation in inoculated 3xTg AD mice. T22-positive high-molecular-weight TauO were successfully enriched from all groups, where rpAD TauO exhibited compact, densely packed oligomers under TEM and the highest phosphorylation at pS396 and pS422, exceeding both spAD and controls (*p* ≤ 0.0327). In 3xTg mice, pS396 showed an early increase followed by a late decline, consistent with dynamic shifts in tau solubility during disease evolution. Brain-derived TauO from spAD and rpAD, but not recombinant tau monomers or control-derived TauO, significantly reduced SH-SY5Y cell viability. Proteomic profiling identified 2388 TauO-associated proteins, including a shared 556-protein core and a striking expansion of rpAD-unique interactors (*n* = 1101). In controls and spAD, the core TauO interactome was enriched for translation, proteostasis, mitochondrial respiration, and vesicle-trafficking pathways, whereas these modules were absent in rpAD. Instead, rpAD TauO showed selective enrichment of aldehyde detoxification, amino-acid and carbon metabolism, and actin-regulatory modules, alongside increased association of SERPINA1, ALDH9A1, MAPRE3, DPYSL2, DPYSL3, and NFASC and reduced coupling to mitochondrial (MRPL17) and complement (C9) components. These convergent structural, post-translational, toxic, and interactome changes indicate that rpAD is defined by a biochemically distinct TauO species embedded in a metabolic and cytoskeleton-focused network, providing a mechanistic framework for its aggressive clinical course and a basis for subtype-specific biomarker and therapeutic strategies.

**Supplementary Information:**

The online version contains supplementary material available at 10.1007/s00401-026-02998-4.

## Introduction

Alzheimer’s disease (AD), the most common neurodegenerative disorder and the leading cause of dementia worldwide, is characterized by progressive cognitive impairment and extensive neuronal loss. The pathological hallmarks of AD are extracellular β-amyloid (Aβ) plaques and intracellular neurofibrillary tangles (NFTs) composed of hyperphosphorylated tau protein [2, 41, 42]. In recent years, the accumulation of Aβ plaques and subsequent NFT growth has been shown to be responsible for downstream synaptic dysfunction and neuronal loss, indicating the onset and progression of AD-associated symptoms [32, 42].

Since the postulation of the amyloid cascade hypothesis in the 1990s, the notion of direct Aβ-driven toxicity has dominated the field. However, the primary clinical and pathological findings have been questioned over the years. Notably, Aβ burden does not correlate well with disease severity or progression, and ~ 30% of elderly individuals harbor substantial Aβ pathology without cognitive impairment [24]. Under pathological conditions, tau, a microtubule-associated protein, becomes hyperphosphorylated and undergoes conformational changes that promote self-association into oligomers, paired helical filaments (PHFs), and NFTs [14].

Striking biochemical diversity exists in the forms of soluble, oligomeric, and seed competent hyperphosphorylated tau in AD patients. In addition, some posttranslational modification sites (PTMs) are associated with the increased seeding activity of tau, leading to worse clinical outcomes [5, 13, 36]. Among tau species, tau oligomers (TauO) are increasingly recognized as the most neurotoxic form, as they mediate synaptic dysfunction, axonal transport impairment, and neuron loss in both in vitro and in vivo models [44]. TauO also exhibit prion-like properties, propagating between cells and promoting further aggregation of native tau [15, 31].

The multiple tau biochemical states and strains may explain the clinical heterogeneity in AD phenotypes. Slowly progressive AD (spAD) and rapidly progressive AD (rpAD) differ not only in their clinical trajectory but also in their molecular features, including tau PTMs and conformational states [24].

Emerging data suggest that tau conformers may form distinct strains with unique pathogenic properties, akin to prions [46]. Subtype-linked differences in soluble and seed-competent tau assemblies have been described in rpAD, where distinct conformers were associated with more aggressive clinical trajectories [13, 27]. More recently, Perbet et al. [37] extended these observations in an independent cohort and provided additional biochemical characterization of subtype-specific tau assemblies. The present study builds on this framework by systematically comparing T22-enriched brain-derived tau oligomers from spAD and rpAD with respect to ultrastructure, phosphorylation state, toxicity, and proteomic interactome composition. We examined their morphology via transmission electron microscopy (TEM), assessed tau phosphorylation patterns in frontal cortex lysates, and characterized their copurified proteome via quantitative mass spectrometry. Our goal was to perform a clinicopathological correlation and identify whether TauO in rpAD differs structurally or molecularly from that in spAD and control brains, potentially offering mechanistic insight into their more aggressive clinical course.

## Methods

### Ethics statement

Frontal cortex tissue samples were collected postmortem from individuals with spAD (*n* = 5), rpAD (*n* = 5) and from age-matched nondemented controls (*n* = 5). Tissues were sourced from the Institute of Neuropathology Brain Bank (HUB-ICO-IDIBELL Biobank) and the Biobank of Hospital Clinic-IDIBAPS, Spain. Additional control samples were obtained from the Department of Neuropathology at the University Medical Center Göttingen, Germany. All procedures complied with national legislation and institutional protocols and were approved by the relevant ethics committees (Spain: HUB-ICO-IDIBELL Biobank, Hospital Clinic-IDIBAPS; Germany: University Medical Center Göttingen, protocols Nr. 1/11/93 and Nr. 9/6/08).

### Patient cohorts and spAD/rpAD subtype characterization

Patient selection and neuropathological assessments followed previously established protocols [51]. All AD patients (spAD and rpAD) exhibited advanced neurofibrillary pathology (Braak stage > V) and were free of coexisting neurodegenerative conditions. Within the rpAD cohort, three of five cases were classified as Braak stage VI, whereas none of the spAD cases reached Braak VI (Supplementary Table 1S). Diagnosis was confirmed through standardized neuropathological evaluation of 25 brain regions, including the cerebral cortex, thalamus, diencephalon, cerebellum, and brainstem, as described previously [22]. Histological techniques included hematoxylin and eosin staining, Klüver–Barrera staining, and immunohistochemistry for β-amyloid, phosphorylated tau, GFAP, alpha-synuclein, TDP-43, ubiquitin, p62, and microglial markers. All rpAD patients fulfilled contemporary diagnostic criteria [40]. Rapidly progressive Alzheimer’s disease (rpAD) was defined based on the rate of cognitive decline, consistent with criteria proposed by Schmidt et al. (2011), [40] as a Mini-Mental State Examination (MMSE) score decrease of ≥ 6 points per year [45]. This operational definition is broadly consistent with prior studies that defined rpAD based on accelerated clinical decline and shortened survival [27, 37], although exact duration thresholds vary slightly across cohorts.

The full Braak and Thal staging details are provided in supplementary Table 1S.

### Cohort and assay allocation

TauO were isolated from 15 patients (control *n=5,* spAD *n=5,* rpAD *n=5).* Western blot PTM assessment, TauO isolation, and TEM were performed on all 15 samples. For discovery proteomics, the eluate per case was finite, and material was allocated prospectively across assays; at the time of LC–MS acquisition, we therefore included the samples with sufficient remaining eluate, yielding control *n=3,* spAD *n=5,* and rpAD *n=3* (rpAD1, rpAD2, and rpAD4; Table S1). All proteomics samples were prepared with equalized input and processed under identical buffers with matched IgG controls. Beads-only immunoprecipitations were not performed.

### Tissue homogenization and protein extraction for TauO

Frozen frontal cortex tissues were homogenized at a 1:3 (w/v) ratio in ice-cold phosphate-buffered saline (PBS) supplemented with a protease inhibitor cocktail (1 tablet per 50 mL, Roche). Homogenization was performed via a mechanical tissue lyser (Qiagen) to ensure complete cellular disruption. The lysates were subsequently centrifuged at 9279×*g* for 10 min at 4 °C. The resulting supernatants (the PBS-soluble fraction) were collected and aliquoted for subsequent biochemical analyses.

### Western blot analysis of TauO

Western blotting was performed to confirm the presence of high-molecular-weight tau oligomers in both total lysates and immunoprecipitated fractions. The samples were diluted in 4 × Laemmli sample buffer (Bio-Rad) without boiling to preserve oligomeric structures. Proteins were separated via NuPAGE on 4–12% Bis–Tris precast gels (Invitrogen) in 1 × MOPS SDS running buffer at 80 V for 10 min followed by 120 V for ~ 1 h. Proteins were transferred to nitrocellulose membranes (0.45 µm, GE Healthcare) via wet transfer (97 V, 1 h, 4 °C). The membranes were blocked in 5% BSA and probed overnight at 4 °C with primary T22 antibody (1:1000) followed by HRP-conjugated anti-mouse secondary antibody. Detection was performed via enhanced chemiluminescence (ECL) and imaging via a ChemiDoc imaging system (Bio-Rad).

### Immunoprecipitation and isolation of TauO

TauO were enriched from PBS-soluble brain lysates via immunoprecipitation using the tau oligomer-specific antibody T22 (Millipore). Tosyl-activated magnetic Dynabeads (Thermo Fisher Scientific) were conjugated with 20 µg of T22 antibody (1.0 mg/mL) in 0.1 M borate buffer (pH 9.5) by overnight incubation at 37 °C. The beads were washed in 0.2 M Tris buffer (pH 8.5) containing 0.1% BSA to remove unbound antibody and block nonspecific binding sites.

The PBS-soluble lysates were incubated with the antibody-conjugated beads under gentle rotation for 1 h at room temperature. After incubation, the beads were washed 3 × in PBS to eliminate unbound material. Bound TauO was eluted with 0.1 M glycine (pH 2.8), and the eluate was immediately neutralized with 1 M Tris–HCl (pH 8.0). The eluted fraction was then concentrated and buffer-exchanged via Microcon centrifugal filters (25 kDa MWCO, Millipore) at 14,000 × g for 25 min at 4 °C. Finally, the TauO protein was resuspended in PBS and quantified via a BCA protein assay (Thermo Fisher).

### Transmission electron microscopy (TEM)

For negative staining of TauO for electron microscopy, 10 µL of the TauO sample mixture was applied to a paraffin film. A glow-discharged carbon-coated copper grid (400 mesh) was then placed onto the drop to allow adsorption of the sample. Subsequently, 10 µL of 0.25% glutaraldehyde was added to the drop and incubated for 1 min to fix the sample. The grid was washed briefly three times by dipping in PBS to remove excess fixative. Next, the grid was incubated for 30 s with 2% uranyl acetate stain. Importantly, no washing was performed after uranyl acetate incubation; instead, excess stain was carefully wicked off, and the grid was air-dried before further analysis. Morphological analysis of TauO was performed via transmission electron microscopy. The stained grids were examined to assess features such as size, shape, circularity, and the presence of electron-dense aggregates in TauO derived from control, spAD, and rpAD samples.

### Cell culture and treatment

Cells were maintained in T75 flasks with DMEM supplemented with 10% FBS and 1% penicillin–streptomycin. At 70–90% confluency, cells were trypsinized, washed with 1 × PBS, and seeded into 96-well plates at a density of 1 × 10^4^ cells/well. After incubation for 18–24 h at 37 °C, cells were treated with brain-derived TauO from experimental and control groups for 18 h. TauO eluates were used to seed recombinant tau prior to cellular treatment. Eluates from matched IgG immunoprecipitations did not induce detectable oligomerization of recombinant tau by Western blot and were therefore not used as treatment conditions.

### MTS assay

Cell viability was assessed using the MTS assay (Abcam, ab197010) according to the manufacturer’s instructions. Briefly, culture medium was replaced with fresh medium prior to adding MTS reagent [3-(4,5-dimethylthiazol-2-yl)-5-(3-carboxymethoxyphenyl)-2-(4-sulfophenyl)-2H-tetrazolium, inner salt]. Cells were incubated for 1 h at 37 °C to allow the formation of a soluble formazan product by metabolically active cells. Absorbance was measured at 490 nm using a Perkin Elmer Wallac 1420 Victor microplate reader (GMI, USA). Background absorbance from control wells was subtracted from experimental readings to obtain final values.

### Label-free quantification mass spectrometry (LFQ-MS) analysis

TauO fractions were resolved by short-term SDS-PAGE (4–20% Bis–Tris) and subjected to in-gel tryptic digestion. Peptide mixtures were spiked with Biognosys iRT standards and analyzed on a Thermo Exploris 480 Orbitrap using a 22-variable window DIA method (60 min gradient, 250 ng equivalent per run). Each biological replicate was analyzed in technical triplicate.

The data were processed in Spectronaut v19.0 (Biognosys) via the Pulsar search engine against the UniProtKB *H. sapiens* reference proteome (release 08/2023) supplemented with a 53-protein contaminant database. Protein and peptide identification was controlled at a 1% false discovery rate (FDR). Quantification was performed by DIA using up to 6 fragments per peptide and up to 10 peptides per protein, with dynamic retention time alignment, dynamic mass recalibration, and quartile normalization. Spectronaut’s built-in global data imputation was applied to the final results table. For downstream filtering, only proteins supported by ≥ 2 stripped peptides in the experiment-wide report were retained. To minimize nonspecific background, matched isotype IgG immunoprecipitations were performed for each group. Proteins enriched ≥ twofold in TauO (T22 IP) relative to their matched IgG controls (linear space; equivalent to log2FC ≥ 1) were considered copurified with TauO. LFQ intensities (arbitrary MS1-based units) were log2-transformed and z scored for heatmap visualization; for boxplots, data are displayed as log10 LFQ intensities (arbitrary units).

### Humanized 3xTg mouse model of combined Aβ and tau pathology

The triple-transgenic (3xTg) mouse model, originally developed by Oddo et al., carries mutations in the APP, PS1, and tau genes, recapitulating key features of combined Aβ and tau pathology [35]. In this study, 3- to 4-month-old 3xTg mice received stereotactic injections of 20 µL of 10% (w/v) cortical brain homogenate from an AD patient, which was targeted to the thalamus. The homogenate was prepared in PBS and clarified by centrifugation prior to inoculation. Both inoculated (AD) and noninoculated control mice were sacrificed at 3, 6, 9, and 12 months post-injection. After decapitation, the cortical tissues were rapidly collected and snap-frozen in liquid nitrogen for further analysis. A sample size of *n* = 4 per group was chosen on the basis of practical considerations and precedent from prior 3xTg studies.

### Gene ontology (GO) term analysis

Functional enrichment and network analyses were performed in Metascape (Homo sapiens). Input lists were derived from tau-oligomer copurified brain proteomes and included: (i) unique sets per condition (Control-only, spAD-only, rpAD-only, shared spAD-rpAD), and (ii) where indicated, core per-condition lists defined purely by identification confidence (proteins with ≥ 2 unique peptides within a condition, independent of abundance). Gene identifiers were supplied as HGNC symbols. For all analyses, the background was the union of all quantified proteins detected in the experiment, to control for mass-spectrometry detection bias. Enrichment was run against GO Biological Process, KEGG, Reactome, and CORUM with Metascape defaults (minimum overlap = 3, *p*-value cutoff = 0.01; Benjamini–Hochberg FDR applied by Metascape). Redundant term clusters were consolidated using Metascape’s default hierarchical reduction. Protein–protein interaction (PPI) modules were identified with MCODE using Metascape defaults (node degree and score thresholds as provided; network size bounds 3–500) on the Physical Core interactome. Disease enrichment used DisGeNET within Metascape. Outputs included heatmaps of enriched terms and PPI networks colored by MCODE cluster; exported PDFs/CSVs were used for Figure assembly and reporting. All analyses were run with the same organism setting (Homo sapiens) and the same custom background to ensure comparability across lists.

### Statistical analysis

Statistical analysis for mass spectrometry data was performed using Python (3.11.9), and GraphPad Prism (version 9). Libraries used in the Python included pandas, matplotlib, and seaborn. Group comparisons were performed in GraphPad Prism v9. For two-group comparisons, Welch’s t test was applied. Statistical significance was defined as *p* < 0.05 on the basis of log2-transformed LFQ intensities.

## Results

Given the clinical and pathological heterogeneity between spAD and rpAD, we explored whether TauO isolated from these subtypes and control brains exhibit distinct structural and molecular characteristics. To explore the molecular differences in tau oligomers across AD subtypes, we conducted a multistep analysis integrating biochemical, ultrastructural, and proteomic approaches.

### Distinct patterns of Tau oligomers in AD subtypes

**Fig. 1:**
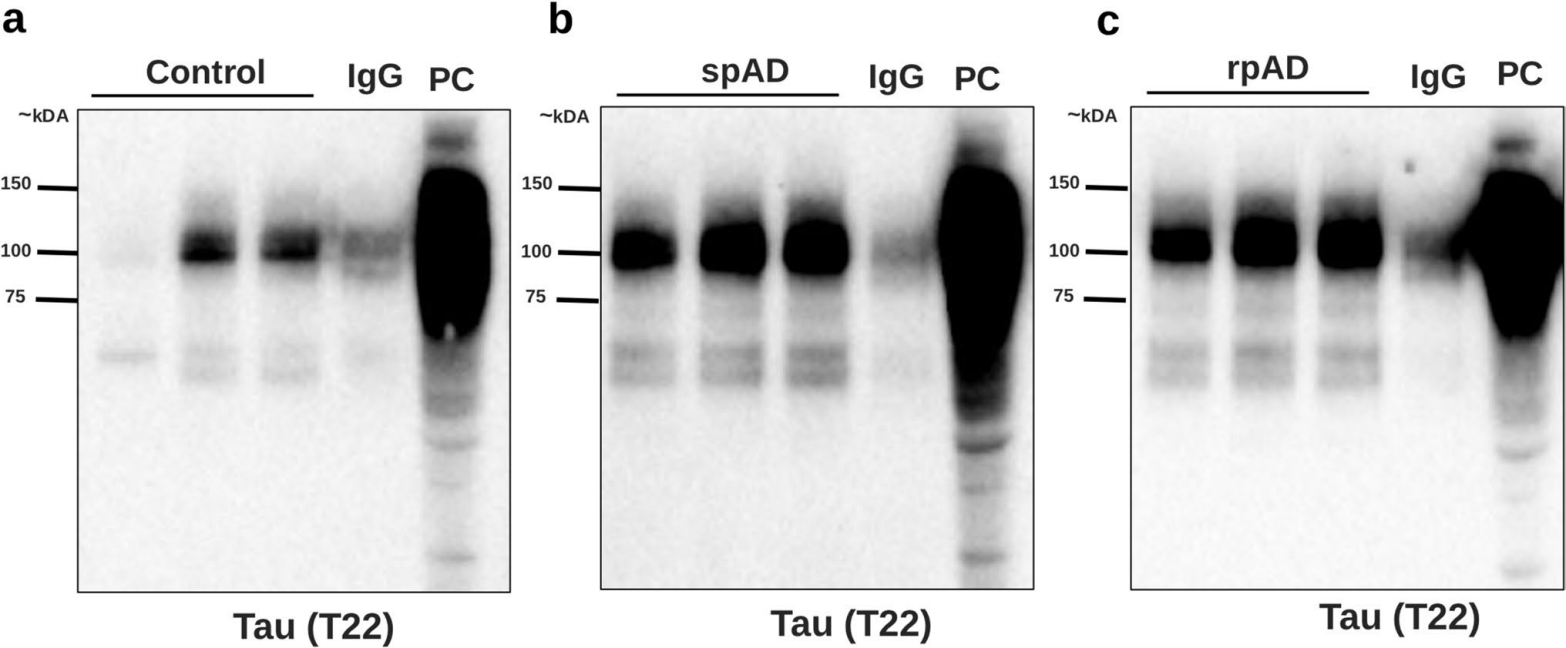
Western blot analysis of isolated TauO in control, spAD, and rpAD brain samples (frontal cortex) via the T22 antibody. TauO were immunoprecipitated from human brain homogenates and probed with a T22 antibody. HMW TauO particles were successfully isolated from all three groups. Representative immunoblots showing tau oligomer profiles in control (**a**), spAD (**b**), and rpAD (**c**) samples. IgG lanes represent isotype IgG negative controls immunoprecipitation, and PC lanes correspond to the positive control. Representative blots from 3 of 5 analyzed samples (*n* = 15 (5 control, 5 spAD, 5 rpAD)). Uncropped blots presented in supplementary Fig. 3S

Western blotting of PBS-soluble brain lysates with the oligomer-selective antibody T22 revealed high-molecular-weight (HMW) tau species across groups, with variable intensity in the control group and more prominent ~ 150 kDa bands in the spAD group (Supplementary Fig. 1S). rpAD showed a distinct but overall less intense HMW pattern. Following T22 immunocapture, HMW TauO was confirmed in eluates from the control, spAD, and rpAD groups (Fig. 1), indicating successful enrichment.

### Tau oligomer morphology differs across disease subtypes

**Fig. 2:**
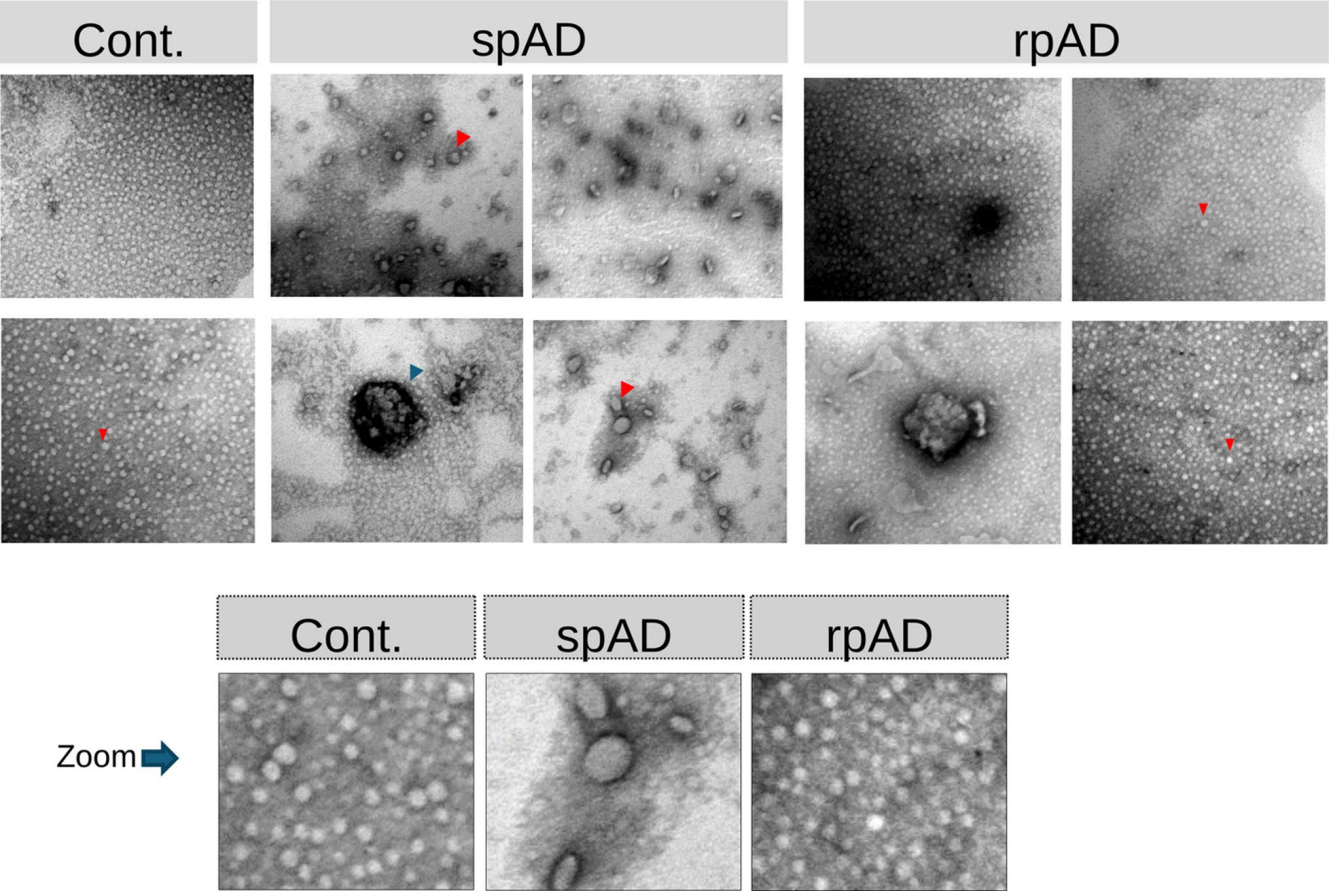
Negative-stain TEM of endogenous T22-enriched tau oligomers from control, spAD, and rpAD frontal cortex. Representative micrographs from T22-enriched soluble fractions (*n* = 5 per group). Control samples show predominantly small spherical particles with infrequent annular structures (red arrowheads). spAD samples display a broader range of particle morphologies, including spherical, annular (red arrowheads), and irregular aggregated forms, as well as occasional membrane-like material (blue arrowhead). rpAD samples show abundant, densely packed small spherical oligomers with additional annular and aggregated species. Bottom panels show zoomed-in regions from representative images. Scale bars = 100 nm

Negative-stain TEM of T22-enriched soluble fractions revealed clear qualitative differences in oligomer appearance across groups (Fig. 2). Control samples showed the lowest oligomer abundance. Many fields contained no visible particles, and representative fields displayed predominantly small, uniformly spherical oligomers with only rare hollow-centered (annular) structures and minimal aggregation. spAD samples exhibited the greatest morphological diversity, including small spherical particles, more frequent annular oligomers, irregular electron-dense aggregates, and occasional membrane-like or vesicular structures. rpAD samples also contained spherical, annular, and aggregated particles; however, their fields were characterized by high oligomer abundance and dense particle clustering, with a visually more uniform population of small round oligomers and fewer distinct structural subclasses compared with spAD. The bottom row shows zoomed-in regions highlighting representative particle morphology in each group. Importantly, no fibrillar, paired helical filament-like, or filamentous tau structures were observed in T22-enriched fractions from any group, consistent with enrichment of prefibrillar oligomeric species.

### Evaluation of tau post-translational modifications (PTMs) in AD subtypes

**Fig. 3:**
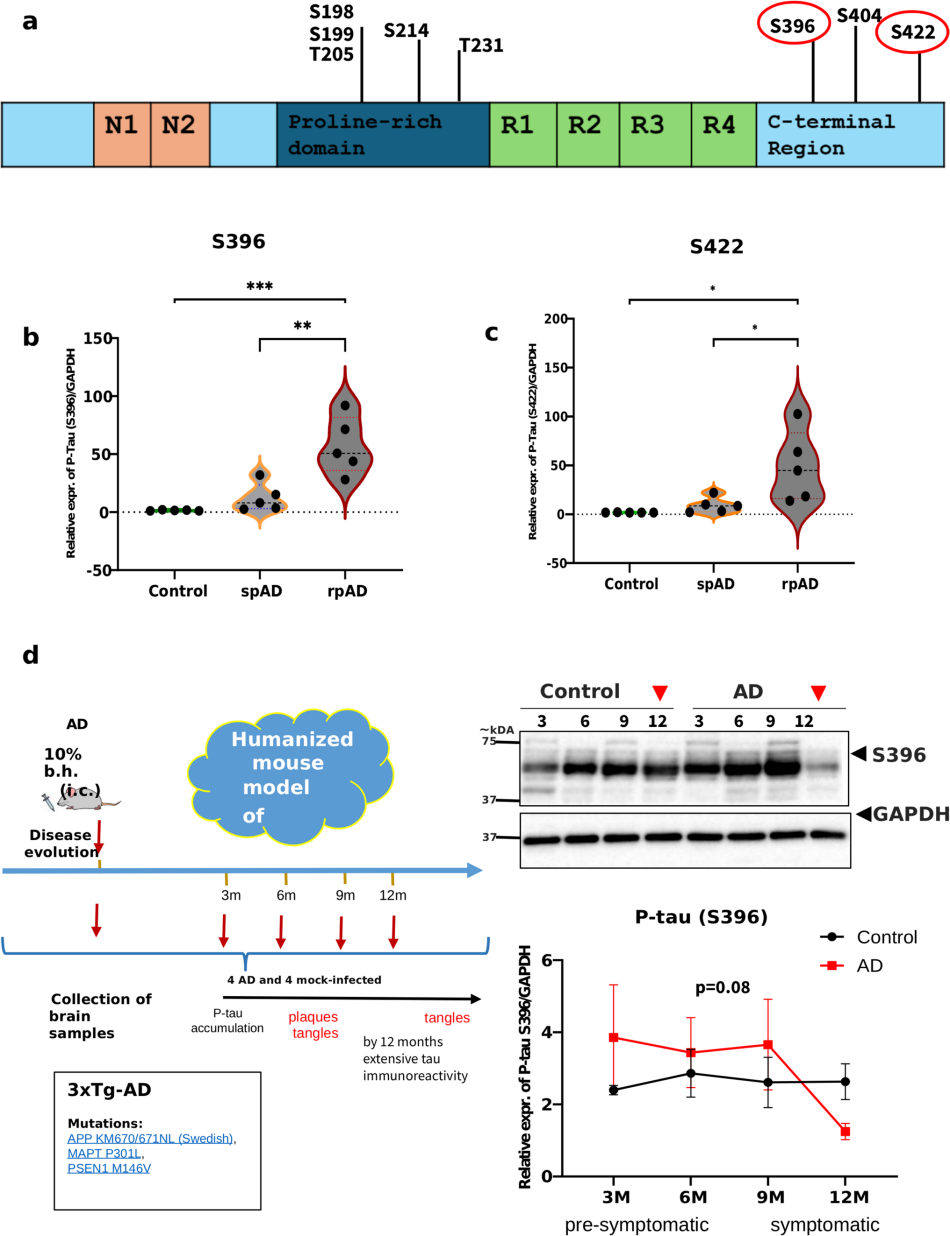
Tau PTMs in control, spAD, and rpAD strains and validation in a 3xTg mouse model. **a** Schematic of tau protein domains indicating the phosphorylation sites examined; sites showing significant differences are highlighted in red circles. **b**, **c** Violin plots of the pS396 and pS422 intensities in the control (*n* = 5), spAD (*n* = 5), and rpAD (*n* = 5) groups. **d** Western blot validation of pS396 expression in cortical tissue from 3xTg AD mice inoculated with AD brain homogenate (*n* = 4 per time point) compared with noninoculated controls (*n* = 4). GAPDH served as a loading control. One-way ANOVA was used for human groups (**b**, **c**), and unpaired t tests with multiple-comparison correction were applied at each time point in mice (**d**). *p < 0.05, **p < 0.01, ***p < 0.001. Uncropped blots presented in supplementary Fig. 3S

Tau oligomerization is strongly influenced by PTMs, particularly phosphorylation, which disrupts microtubule binding and exposes aggregation-prone domains. To assess potential subtype-specific differences, we screened multiple tau phosphorylation sites in urea/thiourea lysates. A schematic of the tau domains and epitopes examined is shown in Fig. 3A. Across the sites analyzed (Tau-5, S198, S199, T205, T231, and S404), no significant group differences were detected (Supplementary Fig. 2S). In contrast, phosphorylation at S396 and S422 displayed the most marked alterations (Fig. 3b, c). The level of phosphorylation at S396 did not differ significantly between spAD patients and controls, but rpAD was markedly greater than both spAD patients (*p* = 0.0021) and controls (*p* = 0.0004). Similarly, phosphorylation at S422 was not significantly altered in spAD versus controls, yet rpAD exhibited significantly elevated levels compared with both groups (*p* = 0.0327 vs spAD; *p* = 0.0125 vs control). Controls exhibited minimal phosphorylation at these epitopes, which was consistent with physiological tau regulation. Hyperphosphorylation at these sites in AD may destabilize microtubule binding and facilitate tau oligomer formation, in line with previous mechanistic studies [48].

### Age-dependent changes in S396 phosphorylation in 3xTg mice

To investigate the temporal regulation of pS396, we examined cortical lysates from 3xTg AD mice inoculated with AD brain homogenates (Fig. 3d). Immunoblotting revealed the progressive accumulation of pS396 tau at early time points (3–9 months post-inoculation), which was consistent with the development of tau pathology. By 12 months, the pS396 levels had declined relative to earlier peaks. This reduction may reflect sequestration of hyperphosphorylated tau into insoluble aggregates or loss of tau-expressing neurons with disease progression [7]. Similar late-stage declines in soluble phosphorylated tau have been described in other AD models, supporting the concept of a dynamic shift in tau solubility during disease evolution [11].

### Brain-derived TauO induces toxicity in a neuronal cell model

**Fig. 4:**
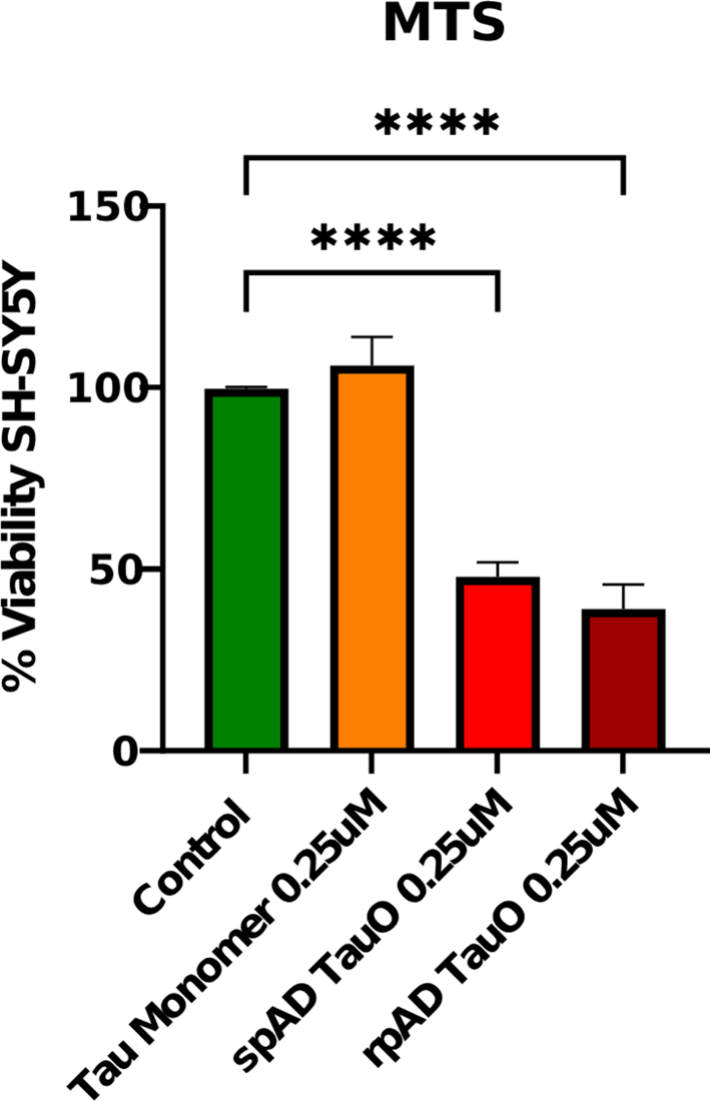
Cytotoxic effects of brain-derived TauO in SH-SY5Y cells. MTS assay quantification of cell metabolic activity following exposure to 0.25 μM recombinant tau monomers (orange) or brain-derived TauO (red = spAD; dark red = rpAD). Control (green) shows maximal viability. TauO treatment significantly reduced the MTS signal relative to that of the controls (*p* < 0.0001 for spAD; *p* < 0.0001 for rpAD). The viability of the rpAD TauO group tended to be lower than that of the spAD TauO group (*p* = 0.27). The bars represent the means ± SEMs.

To evaluate the bioactivity of brain-derived TauO, SH-SY5Y neuroblastoma cells were treated with recombinant tau preincubated with TauO isolated from control, spAD, and rpAD brain homogenates. Successful seeding and aggregation of recombinant tau were verified by T22 immunoreactivity, confirming that all preparations retained oligomeric competence. In contrast to recombinant tau, monomers did not affect cell viability; exposure to 0.25 μM TauO from both spAD and rpAD samples caused a marked reduction in the MTS signal (Fig. 4). The decrease in metabolic activity was more pronounced for rpAD TauO, showing a trend toward greater toxicity compared with spAD TauO, although this difference did not reach statistical significance (*p* = 0.27). This graded pattern from no effect in monomer treated to clear toxicity in spAD and the strongest response in rpAD suggests a hierarchy of bioactivity among human brain-derived TauO species. These findings align with previous reports describing enhanced neurotoxicity and seeding capacity of soluble tau assemblies derived from AD brains [16, 29].

### Comparative mapping of proteins copurified with TauO

**Fig. 5:**
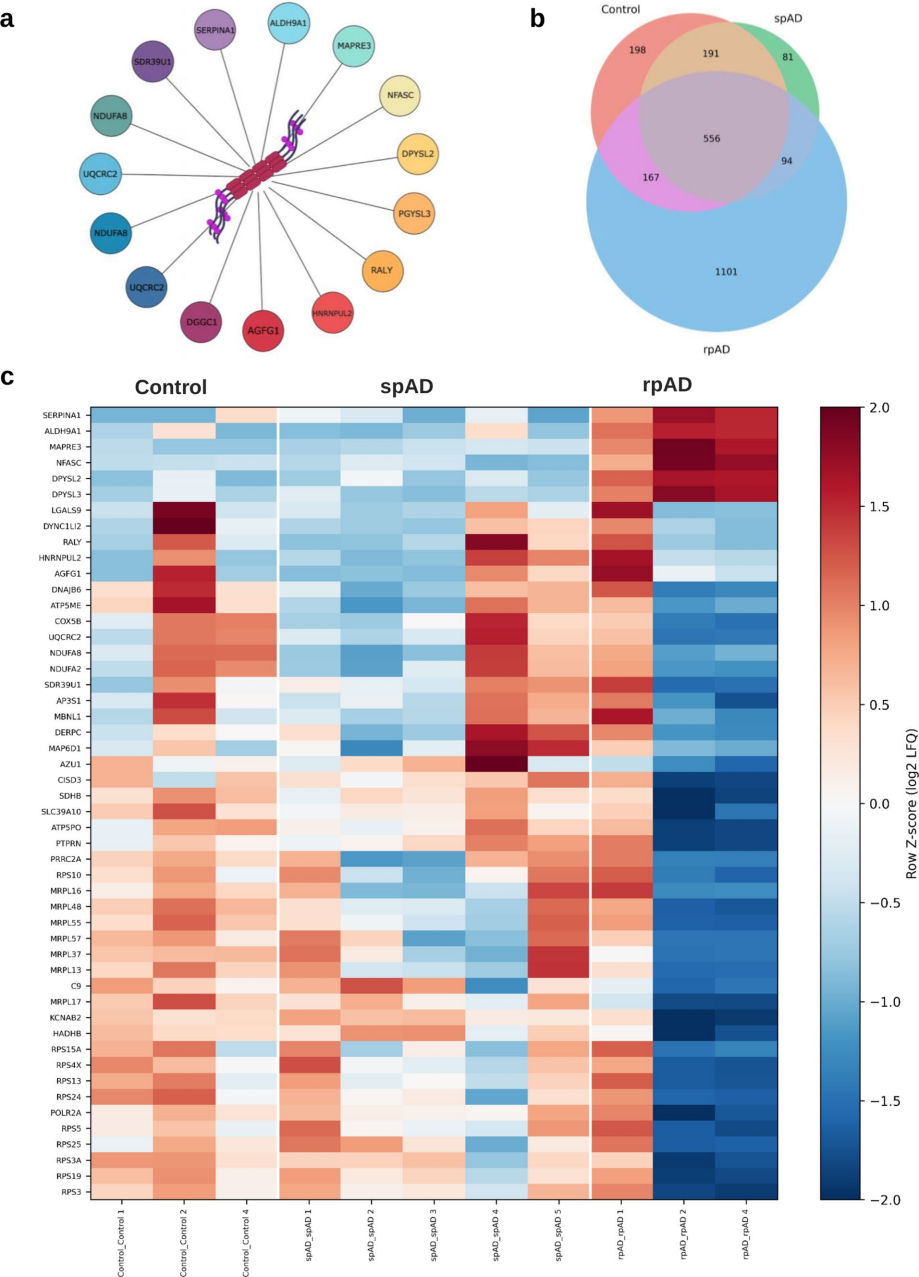
Core tau-oligomer (TauO)-associated proteins identified across disease groups. **a** Representative schematic showing selected proteins detected in TauO-immunoprecipitated fractions. Proteins are depicted as colored nodes surrounding TauO (center) to illustrate the diversity of copurifying interactors. **b** Venn diagram summarizing overlap among proteins retained under uniform inclusion criteria (≥ 2 peptides; ≥ twofold enrichment versus IgG control). Counts: Control-only = 198; spAD-only = 81; rpAD-only = 1101; shared across all = 556; pairwise overlaps as indicated. **c** Heatmap of log₂-transformed, row Z-scored LFQ intensities for the core set of TauO-associated proteins across Control, spAD, and rpAD samples. Each column represents an individual case; rows correspond to quantified proteins. The color scale reflects relative abundance within each protein (*n* = 11; Control = 3, spAD = 5, rpAD = 3). Together, the panels depict group-specific variation in TauO-associated proteomes, highlighting extensive expansion of unique interactors in rpAD alongside quantitative divergence in shared core components

To characterize the subtype-specific TauO proteome, we used label-free quantitative mass spectrometry (Fig. 5a). 2388 proteins were identified across all the groups. The complete list of proteins identified in TauO coimmunoprecipitates and corresponding quantitative values are provided in Supplementary Table 2. Venn diagram analysis revealed 556 proteins shared among the control, spAD, and rpAD groups, likely representing a copurified proteome (Fig. 5b). Remarkably, the rpAD samples presented the greatest number of uniquely enriched proteins (*n* = 1101), indicating a distinct composition of TauO-associated proteins. The spAD and control samples contained 81 and 198 unique proteins, respectively.

A heatmap further illustrated clear groupwise clustering of the differentially enriched proteins that presented unique protein signatures in rpAD patients (Fig. 5c). Despite the heterogeneity within the patient groups, the rpAD TauO samples presented distinct proteomic profiles, with notable enrichment of proteins such as SERPINA1, ALDH9A1, MAPRE3, NFASC, DPYSL2 and DPYSL3, and depletion of proteins such as MRPL17 and C9. Differentially abundant proteins identified across group comparisons are listed in Supplementary Table 4.

### Selective loss of mitochondrial and translational signatures within the core TauO-associated proteome in rpAD

**Fig. 6:**
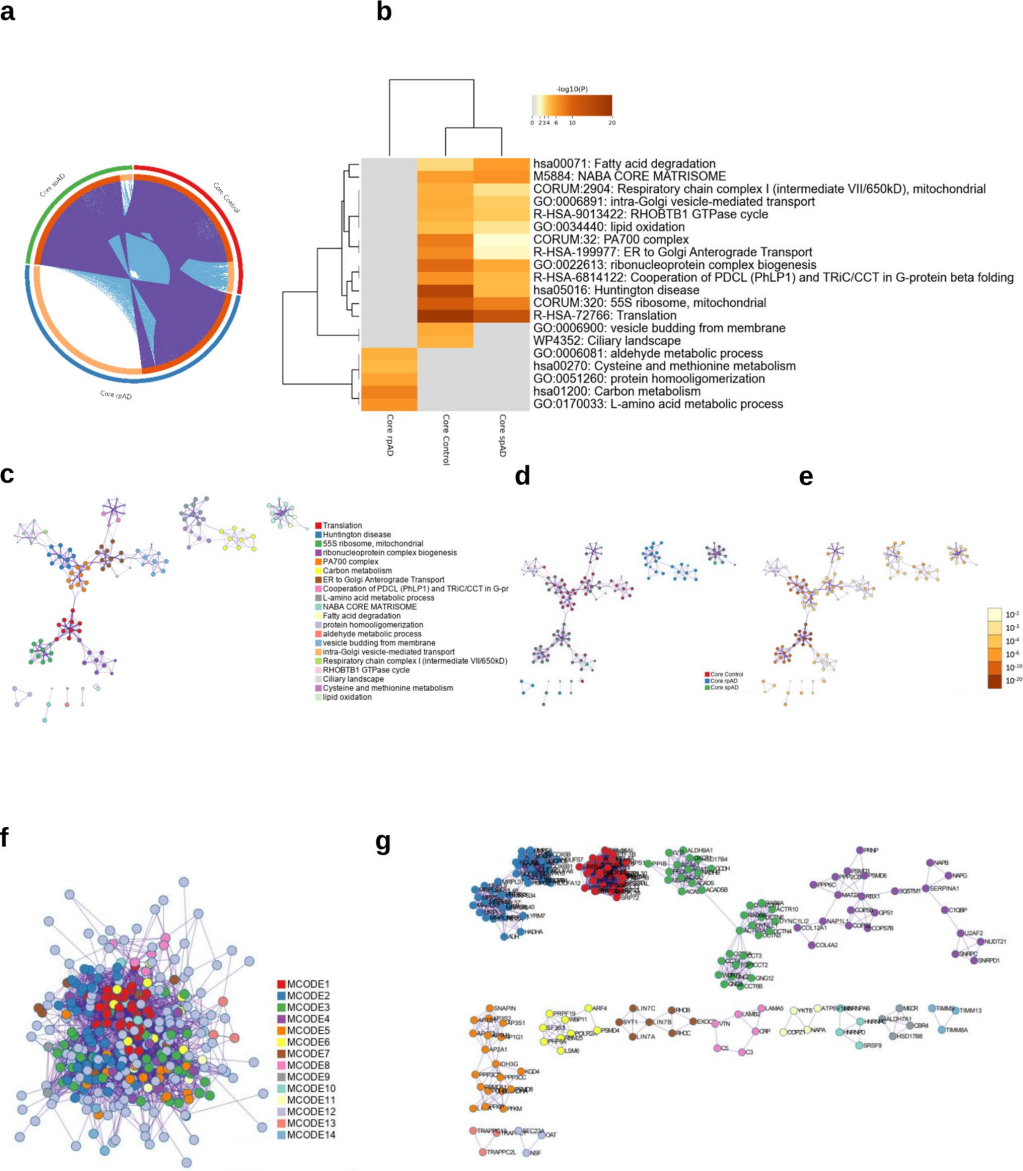
Metascape analysis of the core TauO-associated proteome across control, spAD, and rpAD groups. **a** Circos plot showing the overlap between the core TauO-associated proteins identified in control, spAD, and rpAD groups. Each outer ring represents one condition, and ribbons indicate shared proteins. A large rpAD-specific sector indicates condition-dependent divergence within the core interactome. **b** Heatmap of enriched GO/Reactome pathways (-log₁₀*p*) derived from the core TauO-associated protein sets. control and spAD show enrichment across mitochondrial, proteostasis, vesicle trafficking, and matrisome-related pathways, whereas rpAD displays enrichment restricted to a small set of metabolic processes. **c** Enrichment network in which each node represents an enriched GO/Reactome term. Node size is proportional to the number of input genes annotated to the term, and node color denotes its Metascape-defined cluster identity. Terms with similarity > 0.3 are connected by edges, with thicker edges indicating stronger similarity. **d** The same enrichment network as in **c**, now color-coded by statistical significance (− log_10_*p*). Darker colors denote more significant enrichment. Highly significant clusters correspond to translation, ribosome organization, vesicle trafficking, and mitochondrial pathways enriched in control and spAD. **e** Protein–protein interaction (PPI) network of all core TauO-associated proteins, with node coloration indicating their group of origin (control, spAD, rpAD). The network reflects the shared vs. condition-specific contributions to each connected module. **f** MCODE-clustered PPI network, in which densely connected neighborhoods are assigned distinct colors representing functionally coherent protein modules. **g** Representative MCODE subnetworks, showing modules enriched for translation/ribosome assembly, mitochondrial and metabolic processes, and vesicle-trafficking components

Metascape analysis of the core TauO-associated proteomes revealed that although the three groups share a substantial fraction of interacting proteins, the composition of enriched biological pathways differed sharply across conditions. The Circos plot showed that while a conserved core is present, the rpAD group contributed the largest condition-specific sector, suggesting divergence in pathway-level organization despite partial overlap at the protein level (Fig. 6a). The enrichment heatmap demonstrated a striking functional separation between groups (Fig. 6b). Control and spAD exhibited significant enrichment across the same set of thirteen pathways, including mitochondrial and metabolic functions (fatty acid degradation, lipid oxidation, respiratory chain complex I, carbon metabolism), vesicular trafficking (ER-to-Golgi and intra-Golgi transport, vesicle budding from the membrane), proteostasis-related modules (PA700 complex, ribonucleoprotein complex biogenesis, translation, 55S mitochondrial ribosome, PDCL/TRiC-CCT cooperation), and additional categories such as the NABA core matrisome, etc. In contrast, rpAD showed no enrichment for any of these pathways and instead presented a completely distinct functional profile. Instead, the rpAD core interactome showed enrichment exclusively for five metabolic pathways absent from both control and spAD: aldehyde metabolic process, cysteine and methionine metabolism, protein homooligomerization, carbon metabolism, and l-amino-acid metabolic process. No pathway was shared across all three groups, and neither control nor spAD displayed any uniquely enriched pathway. Network-based visualization of these enriched terms further resolved them into coherent functional modules (Fig. 6c, d). Control and spAD clustered into dense modules corresponding to translation/ribosome/proteostasis, vesicle transport, and mitochondrial metabolism. The rpAD-specific pathways formed a small but clearly separated metabolic module. Coloring by − log10(*p*) highlighted that the shared modules carried the highest statistical support.

Protein–protein interaction analysis with MCODE revealed densely connected subnetworks that mirrored these pathway themes, with large clusters representing translation machinery, mitochondrial proteins, and trafficking complexes in control/spAD, and a smaller, distinct metabolic module in rpAD (Fig. 6e–g). Together, these data demonstrate that the core TauO interactome is structurally shared but functionally divergent, with control and spAD maintaining robust enrichment across proteostasis, mitochondrial, and trafficking modules, whereas rpAD exhibits a complete loss of these signatures and instead shows selective enrichment in a small set of metabolic pathways.

### Unique TauO interactome exhibit distinct functional signatures across groups

**Fig. 7:**
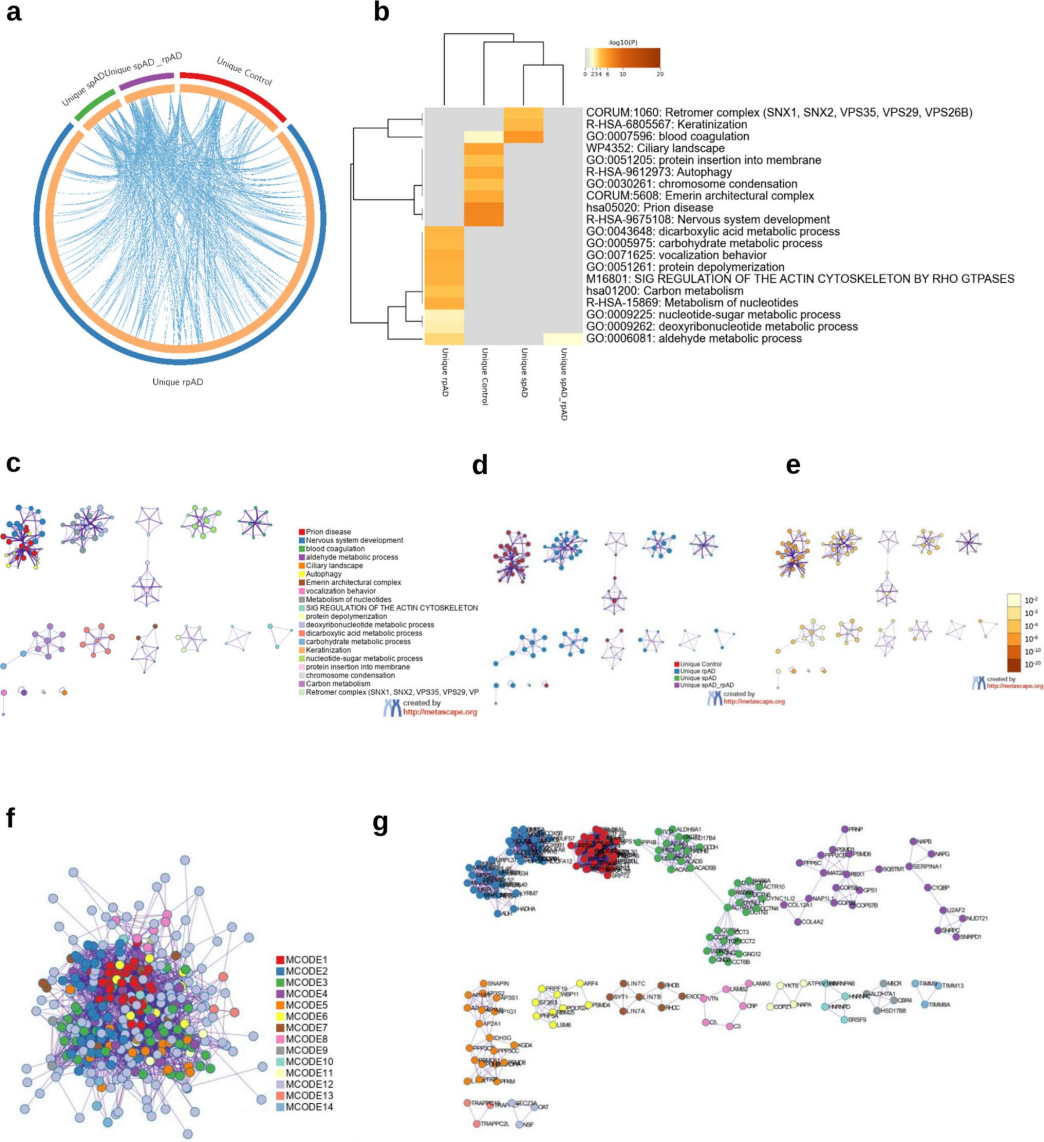
Pathway enrichment and network organization of unique TauO-interacting proteomes. **a** Circos plot showing the overlap between proteins uniquely identified in the control, spAD, and rpAD TauO-immunoprecipitated fractions. Each colored ribbon represents a group, and intersections indicate shared proteins. **b** Heatmap of enriched GO/reactome pathways (-log_10_*p*) derived from proteins unique to each condition. **c** Enrichment network in which each node represents an enriched term. Node size is proportional to the number of input genes annotated to that term, and node color denotes cluster identity. Terms with a similarity score > 0.3 are connected by edges, with thicker edges indicating higher similarity. **d** The same enrichment network as in **c**, but with nodes color-coded by statistical significance (− log_10_*p*). Darker colors indicate more significant enrichment. **e** Protein–protein interaction (PPI) network constructed from all PPI relationships among genes unique to each group. **f** MCODE-clustered PPI network generated from the control-unique protein set, showing densely connected subnetworks. **g** Representative MCODE subnetworks from the control-unique interactome, illustrating modules linked to autophagy, membrane insertion, nuclear architecture, and related processes

To define condition-specific TauO interactomes, we analyzed proteins uniquely copurified with TauO in each group. The Circos plot showed minimal overlap between unique protein sets, indicating that each condition contains a largely distinct complement of TauO-associated proteins (Fig. 7a). Functional enrichment analysis revealed striking differences in pathway composition across groups (Fig. 7b). The rpAD-unique interactome showed the broadest functional representation, with significant enrichment across multiple metabolic categories, including dicarboxylic acid metabolism, carbohydrate metabolism, carbon metabolism, metabolism of nucleotides, nucleotide-sugar and deoxyribonucleotide metabolism, together with protein depolymerization and regulation of the actin cytoskeleton by Rho GTPases (SIG regulation).

The control-unique interactome exhibited enrichment for processes related to autophagy, protein insertion into membranes, chromosome condensation, ciliary functions, the Emerin architectural complex, and terms associated with prion disease and nervous system development. In contrast, the spAD-unique interactome showed a much narrower profile, with enrichment limited to the retromer complex and keratinization. Only a single pathway, blood coagulation, was shared between control and spAD but absent in rpAD, while aldehyde metabolic process represented the only pathway shared between rpAD and spAD.

Network-based pathway visualizations using Metascape further resolved these enrichments into discrete functional modules (Fig. 7c–g). The rpAD-unique terms formed the largest and most densely connected clusters, dominated by metabolic and actin-regulatory modules. Control-unique terms grouped into structural, nuclear, and cilia-associated modules, while the spAD-unique set formed a compact retromer-associated trafficking module. MCODE subnetwork analysis further resolved these enrichment profiles. In the control-unique interactome, major modules reflected translation and proteostasis (MCODE1), mitochondrial translation (MCODE2), chromosome and DNA organization (MCODE3-4), intermediate filament architecture, and ECM/laminin-integrin structural clusters. The rpAD-unique interactome yielded a single metabolically driven module centered on carboxylic acid and nucleotide metabolism, whereas spAD formed a compact retromer/retrograde trafficking module together with a coagulation cluster and a smaller mRNA-processing subnetwork. The shared spAD-rpAD subset produced a single amino-acid catabolic module. These MCODE-defined clusters further support and refine the pathway-level distinctions observed across conditions. Proteins shared between control, spAD, and rpAD groups as well as group-specific proteins are presented in Supplementary Table 3.

## Discussion

Alzheimer’s disease is a clinically heterogeneous disorder, with distinct subtypes, such as spAD and rpAD, that differ in clinical course, neuropathological features, and molecular signatures [40] While spAD progresses over several years, rpAD is characterized by an abrupt cognitive decline and markedly shorter survival [8, 47] The molecular determinants underlying this divergence remain poorly understood.

Here, we examined whether structural, biochemical, or proteomic features of TauO, a key pathogenic intermediate, differ between AD subtypes. We identified multiple convergent features that differentiate rpAD TauO from spAD and control TauO. Recent work has demonstrated that distinct tau conformers and seed-competent assemblies correlate with clinical heterogeneity in AD [13, 27, 37]. Kim et al. identified conformational differences in soluble tau species between rapidly progressive and typical AD, linking these assemblies to differential seeding activity [27]. Perbet et al. further validated subtype-associated biochemical differences in an independent cohort and reported distinct aggregation and stability properties of tau assemblies in rpAD [37]. Our findings are consistent with these observations in that rpAD exhibited a distinct phosphorylation pattern and a markedly divergent interactome profile compared to spAD. While prior studies focused primarily on conformational and seeding properties, the present work extends these findings by demonstrating subtype-specific differences in copurifying protein networks associated with T22-defined tau oligomers.

The rpAD-specific hyperphosphorylation at pS396 and pS422 in frontal cortex PBS-soluble lysates in the present study highlights a soluble tau biochemical state enriched in aggressive AD. This directly parallels Kumar et al.’s [28] MS profiling of PBS-extractable HMW tau seeds from AD frontal/temporal cortex, where analogous proline-rich domain phosphorylations (e.g., pS68/T69/T71, pT205) and overall PTM multiplicity rather than specific sites correlated with seeding potency, preceding full PHF maturation. Wesseling et al. (2020) [49] further contextualize these epitopes as late-stage (Braak V/VI) markers, aligning with our rpAD Braak enrichment [49]. These sites are associated with increased seeding activity and pathological progression in AD models [34, 38] These PTMs may increase the formation and persistence of toxic oligomeric tau species, [3, 13] particularly rpAD.

The absence of these modifications in control TauO, despite detectable oligomeric bands by T22, suggests that phosphorylation rather than mere oligomerization is a key determinant of pathogenicity. In the 3xTg mouse model, we observed a temporal increase and subsequent decrease in pS396 tau levels, consistent with dynamic changes in tau solubility during disease progression [11, 35].

TEM analysis revealed that TauO morphology varied by diagnostic group. Control-derived TauO proteins are small and dispersed, which is consistent with physiological tau assemblies that stabilize microtubules [9]. SH-SY5Y cells treated with control-derived TauO showed no significant decrease in viability, indicating that these oligomers are likely nontoxic. This aligns with prior work showing that brain-derived tau oligomers can share overall size and shape yet differ in seeding and toxic properties depending on conformation and PTM pattern [4], 9.

In contrast, spAD samples contained irregular, vesicle-like structures, suggesting early aggregation events [14, 39] whereas rpAD-derived TauO lacked these vesicle-like assemblies and instead exhibited abundant, small, dense oligomers. This may reflect a distinct aggregation trajectory involving rapid oligomer formation without progression to larger fibrillar structures. Prior studies have shown that specific tau conformers can form structurally distinct “strains” with variable toxicity and propagation potential [26], 46. Our data, therefore, argue that morphology alone is insufficient to define pathological TauO; rather, a combination of PTMs and protein interactors likely tunes oligomer stability, cellular targeting, and toxicity.

Proteomic analyses of detergent-insoluble fractions in Alzheimer’s disease have revealed complex aggregate-associated protein networks extending beyond tau itself. The molecular composition of mature neurofibrillary tangles and detergent-insoluble tau pathology has been characterized by several groups [18, 25, 30]. Building upon these observations, we investigated the Tau interactome to determine which proteins directly associate with tau complexes and how these interactions differ between control and disease conditions. To place the TauO-associated proteome in the context of established tau pathology datasets, we performed cross-dataset comparisons with two independent human AD proteomic studies. First, comparison with the detergent-insoluble brain proteome described by Hales et al., which quantified proteins accumulating in the sarkosyl-insoluble fraction across AD progression, identified 602 overlapping proteins with our T22 coimmunoprecipitated TauO dataset [18]. This overlap included proteins involved in cytoskeletal organization, RNA binding, metabolic processes, and proteostasis, consistent with prior reports that detergent-insoluble pathology engages broad cellular stress networks.

Second, comparison with the phosphorylated tau interactome characterized by Drummond et al., which profiled laser-capture-microdissected neurofibrillary tangles and PHF1-positive tau-associated proteins, revealed 294 overlapping proteins. The shared proteins spanned cytoskeletal regulators, RNA-associated factors, and metabolic enzymes, supporting partial molecular continuity between prefibrillar TauO assemblies and mature phosphorylated tau pathology [12]. Importantly, the incomplete overlap across both datasets indicates that T22-defined TauO represents a distinct, soluble assembly state that shares but does not fully recapitulate the protein environment of detergent-insoluble tau pathology or mature neurofibrillary tangles.

Among individual proteins, several interactors showed increased association with TauO in rpAD relative to control and spAD, highlighting a distinct molecular environment underlying the rapid phenotype. SERPINA1 (α1-antitrypsin), a serine protease inhibitor with established neuroinflammatory relevance, was more enriched in rpAD TauO complexes [1], and its increased enrichment suggests heightened engagement of protease-regulatory or extracellular matrix-modifying pathways. Similarly, ALDH9A1 and related aldehyde dehydrogenases were more abundant in rpAD TauO fractions. Their increased association aligns with the aldehyde-metabolism signature in rpAD and suggests that TauO in this subtype may sequester or dysregulate detoxifying enzymes at sites of metabolic stress. Cytoskeletal regulators DPYSL2 (CRMP2) and DPYSL3 (CRMP4), which modulate axon guidance and microtubule stability and have been implicated in AD, were also elevated in rpAD TauO [21] pointing to enhanced interaction between oligomeric tau and “tau-like” microtubule-associated proteins and potentially amplifying cytoskeletal destabilization in this aggressive clinical variant. NFASC (neurofascin), an axon initial segment and node-of-Ranvier adhesion molecule essential for saltatory conduction, was similarly enriched [52] raising the possibility that rpAD TauO more strongly targets axoglial junctions. Another rpAD-specific increase was observed for MAPRE3 (EB3), a microtubule-plus-end tracking protein that stabilizes dynamic microtubules and interacts with tau [43] suggesting altered regulation of microtubule plus-end dynamics in rpAD. In contrast, MRPL17, a mitochondrial ribosomal protein, and the terminal complement component C9 showed reduced association with TauO in rpAD, implying weakened coupling between TauO and mitochondrial translation and complement-related pathways that may otherwise support metabolic resilience and immune responses [33].

By integrating label-free proteomics with Metascape analysis, we delineated a shared “core” TauO interactome as well as condition-specific unique interactomes. Because the T22 immunoprecipitation captures copurifying rather than strictly direct interactors, altered association likely reflects pathway-level compartmentalization. The core interactome in controls and spAD was dominated by modules related to translation, proteostasis, mitochondrial metabolism, and vesicle trafficking, consistent with earlier tau interactome studies [19].

In striking contrast, the rpAD core interactome showed complete loss of enrichment for these translation, proteostasis, and vesicle-trafficking signatures. Instead, rpAD core TauO showed enrichment restricted to a small set of metabolic pathways, including aldehyde metabolism, cysteine and methionine metabolism, and carbon and amino-acid metabolism. The absence of any pathway jointly enriched across all three groups indicates that, despite overlap at the individual protein level, the functional organization of TauO interactomes diverges sharply in rpAD. One interpretation is that in typical spAD, TauO remains embedded within canonical translation/mitochondrial/trafficking networks that may reflect both physiological tau functions and early stress responses, whereas in rpAD these networks are disengaged or overwhelmed and TauO becomes selectively coupled to metabolic stress modules [6, 23].

Analysis of proteins uniquely copurifying with TauO in each group further sharpened these differences. The control-unique interactome was enriched for autophagy, protein insertion into membranes, nuclear and chromosomal organization, ciliary structures, and pathways associated with nervous system development and prion disease, consistent with a role for tau in vesicle trafficking and cellular quality-control mechanisms [26].

The spAD-unique interactome was more focused, with enrichment concentrated in the retromer complex and retrograde transport pathways, together with keratinization and blood coagulation terms. Retromer dysfunction and endosomal trafficking defects are increasingly recognized as early features of AD and as contributors to both amyloid and tau pathology [10]. The strong retromer signature in spAD-unique TauO suggests that oligomers in typical AD may preferentially engage endosomal/retrograde transport machinery, potentially facilitating propagation along defined neuronal pathways. By contrast, rpAD-unique TauO showed the broadest functional representation, dominated by metabolic pathways (dicarboxylic acid, carbohydrate and carbon metabolism; metabolism of nucleotides and nucleotide-sugars; aldehyde metabolism) and regulation of the actin cytoskeleton by Rho GTPases, together with protein depolymerization. Enrichment of aldehyde metabolism and multiple carbon/energy pathways is notable given accumulating evidence that toxic aldehydes and impaired aldehyde dehydrogenase activity contribute to neuronal death and AD progression [17, 50]. Coupling of rpAD TauO to metabolic enzymes suggests that oligomers in this subtype may directly perturb detoxification and energy metabolism rather than primarily engaging translational or lysosomal machinery. The Rho-GTPase/actin-cytoskeleton signature aligns with reports that tau and tau oligomers remodel actin networks and spine structure, thereby contributing to synaptic failure [1]. Network analysis and MCODE-based PPI clustering further supported these patterns, with controls and spAD dominated by translation/proteostasis, mitochondrial, and trafficking modules, whereas rpAD yielded a compact metabolic module centered on carboxylic-acid and nucleotide metabolism. These network-level differences support a model in which TauO in rpAD is embedded in a qualitatively distinct cellular context dominated by metabolic stress and cytoskeletal remodeling. These alterations support the concept that TauO are not passive aggregates but copurify with functionally relevant proteins, potentially reflecting altered associations that exacerbate cellular stress and degeneration in rpAD [20].

As a cross-sectional postmortem study, our work provides a late-stage snapshot of TauO biology; future longitudinal and mechanistic studies will be required to determine when these subtype-specific interactome differences arise and how they influence disease progression. An additional consideration is that the rpAD cohort was enriched for Braak stage VI cases (3/5), whereas none of the spAD cases reached Braak VI. Because tau pathology in frontal cortex increases substantially at Braak VI [49], regional disease stage may have contributed to the observed biochemical differences. To address this, phosphorylation analyses were examined separately in the two Braak V rpAD cases and revealed the same directional increase in pS396 and pS422 compared with spAD (data not shown), supporting a subtype-associated pattern rather than a purely stage-dependent effect. Due to the limited number of Braak V rpAD samples included in the proteomic analysis, stage-stratified interactome comparisons were not feasible.

In summary, our data demonstrate that TauO derived from rpAD differs from those in spAD at structural, biochemical, and interactome levels. rpAD TauO exhibited compact ultrastructural morphology, elevated disease-associated phosphorylation (pS396 and pS422), and a reorganized protein interaction network characterized by selective enrichment of metabolic and cytoskeletal modules alongside reduced coupling to mitochondrial and complement-related pathways. These findings indicate that TauO in rpAD exists within a distinct molecular environment that may contribute to metabolic vulnerability and accelerated neurodegeneration. While future stage-matched and mechanistic studies are required to determine causality, the present work provides a framework for understanding TauO heterogeneity across AD subtypes.

## Supplementary Information

Below is the link to the electronic supplementary material.

Supplementary file1 (DOCX 1179 KB)

Supplementary file2 (XLSX 606 KB)

Supplementary file3 (XLSX 142 KB)

Supplementary file4 (XLSX 18 KB)

## Data Availability

The mass spectrometry proteomics data have been deposited to the ProteomeXchange Consortium via the jPOST partner repository with the dataset identifier PXD075361. The dataset is available at: https://repository.jpostdb.org/entry/JPST004458.
