# Supplementary material for: A distinct tau oligomer strain defines the molecular and proteomic landscape of rapidly progressive Alzheimer’s disease
Source: Acta Neuropathol. 2026 Mar 18;151(1):27. doi: 10.1007/s00401-026-02998-4 (PMC12999710; doi:10.1007/s00401-026-02998-4)
Supplement: Supplementary file 1 — Supplementary file1 (DOCX 1179 KB) [file 401_2026_2998_MOESM1_ESM.docx]

**A distinct tau oligomer strain defines the molecular and proteomic landscape of rapidly progressive Alzheimer’s disease**

Tayyaba Saleem^a,b*^, Wiebke Möbius^c^, Matthias Schmitz^a,b^, Angela da Silva Correia^a,b^, Carolina Thomas^d,e^, Sezgi Canaslan^a,b^, Peter Hermann^a,b^, Stefan Goebel^a,b^, Saima Zafar^a,b,f^, Elisabeth Root^a^, Christine Stadelmann^d^, Olivier Andreoletti^g^, Michael Hoppert^h^, Tiago Fleming Outeiro^i,j^, Isidre Ferrer^k,^ Neelam Younas^a,b#*^, Inga Zerr^a,b#^

1. Department of Neurology, University Medical Center, Georg-August-Universitäts, Robert-Koch-straße 40, 37075, Göttingen, Germany
2. German Center for Neurodegenerative Diseases (DZNE), Robert Koch-Straße 40, 37075, Göttingen, Germany
3. Department of Neurogenetics, Electron Microscopy City Campus, Max Planck Institute for Multidisciplinary Sciences, Göttingen, Germany
4. Department of Neuropathology, University Medical Center, Göttingen, Germany
5. Paul-Flechsig Institute, Centre for Neuropathology and Brain Research, Leipzig, Germany
6. Biomedical Engineering and Sciences Department, School of Mechanical and Manufacturing Engineering (SMME), National University of Sciences and Technology (NUST), Islamabad, Pakistan
7. UMR INRA ENVT 1225- Interactions Hôte Agent Pathogène–École Nationale Vétérinaire de Toulouse, Toulouse, France
8. Institute of Microbiology and Genetics, Grisebachstr. 8, 37077 Göttingen, Georg-August-University Göttingen, Germany
9. Experimental Neurodegeneration, University Medical Center Göttingen, Waldweg 33, 37073 Göttingen, Germany
10. Translational and Clinical Research Institute, Faculty of Medical Sciences, Newcastle University, Framlington Place, Newcastle Upon Tyne, NE2 4HH, United Kingdom
11. Emeritus Professor, University of Barcelona, Barcelona, Spain

# Shared last authorship

**Supplementary**

##### Tissue homogenization and protein extraction for TauO

Frozen frontal cortex tissues were homogenized at a 1:3 (w/v) ratio in ice-cold phosphate-buffered saline (PBS) supplemented with a protease inhibitor cocktail (1 tablet per 50 mL, Roche). Homogenization was performed via a mechanical tissue lyser (Qiagen) to ensure complete cellular disruption. The lysates were subsequently centrifuged at 9,279 × g for 10 minutes at 4 °C. The resulting supernatants (the PBS-soluble fraction) were collected and aliquoted for subsequent biochemical analyses.

##### Western blot analysis of TauO

Western blotting was performed to confirm the presence of high-molecular-weight tau oligomers in both total lysates and immunoprecipitated fractions. The samples were diluted in 4× Laemmli sample buffer (Bio-Rad) without boiling to preserve oligomeric structures. Proteins were separated via NuPAGE on 4-12% Bis-Tris precast gels (Invitrogen) in 1× MOPS SDS running buffer at 80 V for 10 minutes followed by 120 V for ~1 hour. Proteins were transferred to nitrocellulose membranes (0.45 µm, GE Healthcare) via wet transfer (97 V, 1 hour, 4 °C). The membranes were blocked in 5% BSA and probed overnight at 4°C with primary T22 antibody (1:1000) followed by HRP-conjugated anti-mouse secondary antibody. Detection was performed via enhanced chemiluminescence (ECL) and imaging via a ChemiDoc imaging system (Bio-Rad).

##### Immunoprecipitation and isolation of TauO

Tau oligomers (TauO) were enriched from PBS-soluble brain lysates via immunoprecipitation using the tau oligomer-specific antibody T22 (Millipore). Tosyl-activated magnetic Dynabeads (Thermo Fisher Scientific) were conjugated with 20 µg of T22 antibody (1.0 mg/mL) in 0.1 M borate buffer (pH 9.5) by overnight incubation at 37 °C. The beads were washed in 0.2 M Tris buffer (pH 8.5) containing 0.1% BSA to remove unbound antibody and block nonspecific binding sites.

The PBS-soluble lysates were incubated with the antibody-conjugated beads under gentle rotation for 1 hour at room temperature. After incubation, the beads were washed 3× in PBS to eliminate unbound material. Bound TauO was eluted with 0.1 M glycine (pH 2.8), and the eluate was immediately neutralized with 1 M Tris-HCl (pH 8.0). The eluted fraction was then concentrated and buffer-exchanged via Microcon centrifugal filters (25 kDa MWCO, Millipore) at 14,000 × g for 25 minutes at 4 °C. Finally, the TauO protein was resuspended in PBS and quantified via a BCA protein assay (Thermo Fisher).

### ****Transmission electron microscopy (TEM)****

For negative staining of TauO for electron microscopy, 10 µL of the TauO sample mixture was applied to a paraffin film. A glow-discharged carbon-coated copper grid (400 mesh) was then placed onto the drop to allow adsorption of the sample. Subsequently, 10 µL of 0.25% glutaraldehyde was added to the drop and incubated for 1 min to fix the sample. The grid was washed briefly three times by dipping in PBS to remove excess fixative. Next, the grid was incubated for 30 seconds with 2% uranyl acetate stain. Importantly, no washing was performed after uranyl acetate incubation; instead, excess stain was carefully wicked off, and the grid was air-dried before further analysis. Morphological analysis of TauO was performed via transmission electron microscopy. The stained grids were examined to assess features such as size, shape, circularity, and the presence of electron-dense aggregates in TauO derived from control, spAD, and rpAD samples.


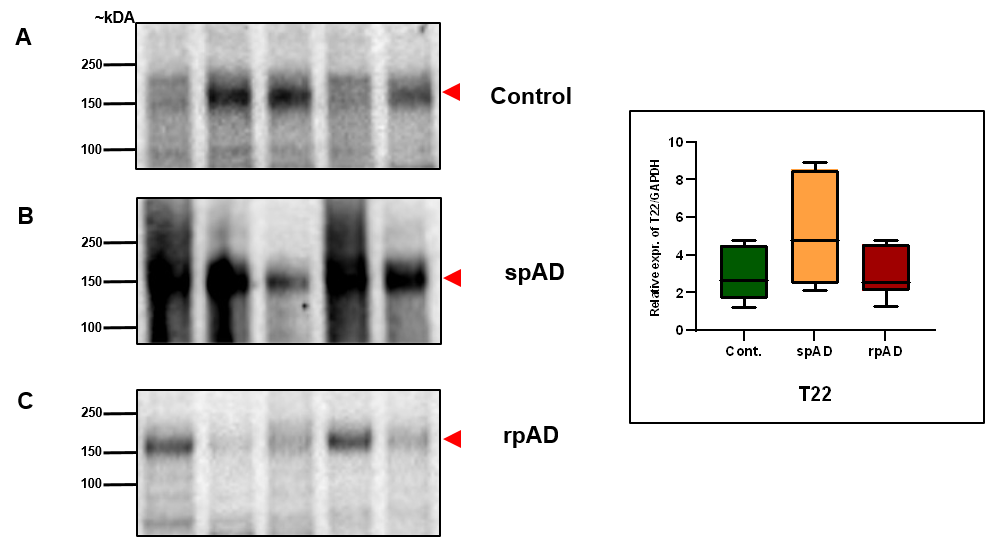


**Fig 1S Western blot analysis of tau species in PBS crude lysates across the control spAD and rpAD groups.** Representative Western blot images (left) display HMW TauO detected in the control (A), spAD (B), and rpAD (C) groups, with the quantification of band intensities shown in the adjacent bar graph (right). Compared with the control and rpAD groups, the spAD group presented an increase in the intensity of HMW tau species. (n=15 (5 control, 5 spAD, 5 rpAD)). Uncropped blots presented in supplementary figure 3S (B)


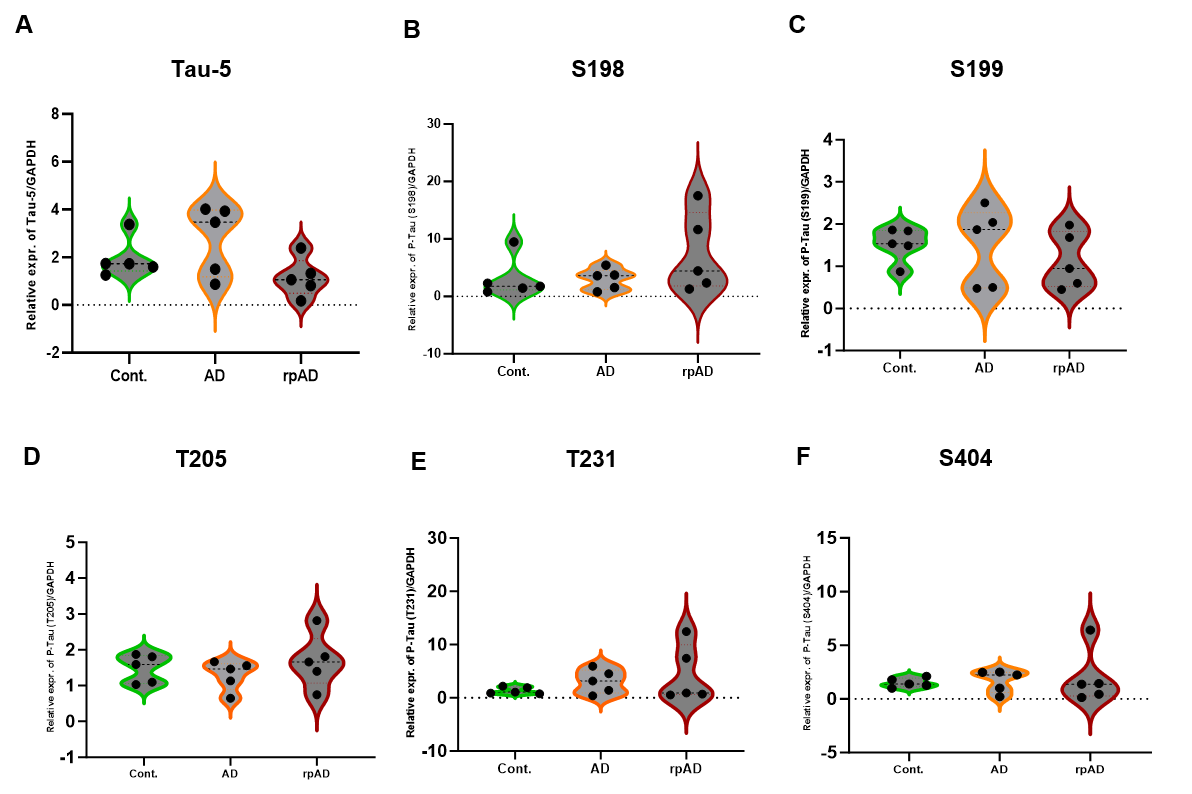


**Fig 2S Phosphorylation of soluble tau at selected epitopes in AD subtypes and controls.**

Violin plots showing Western blot quantification of total tau (A, Tau-5) and phosphorylation at key residues (B, S198; C, S199; D, T205; E, T231; F, S404) in urea thiourea brain lysates from control, spAD, and rpAD patients. No statistically significant differences were observed across groups. Data represent individual cases quantified relative to total protein loading (n=15 (5 control, 5 spAD, 5 rpAD))


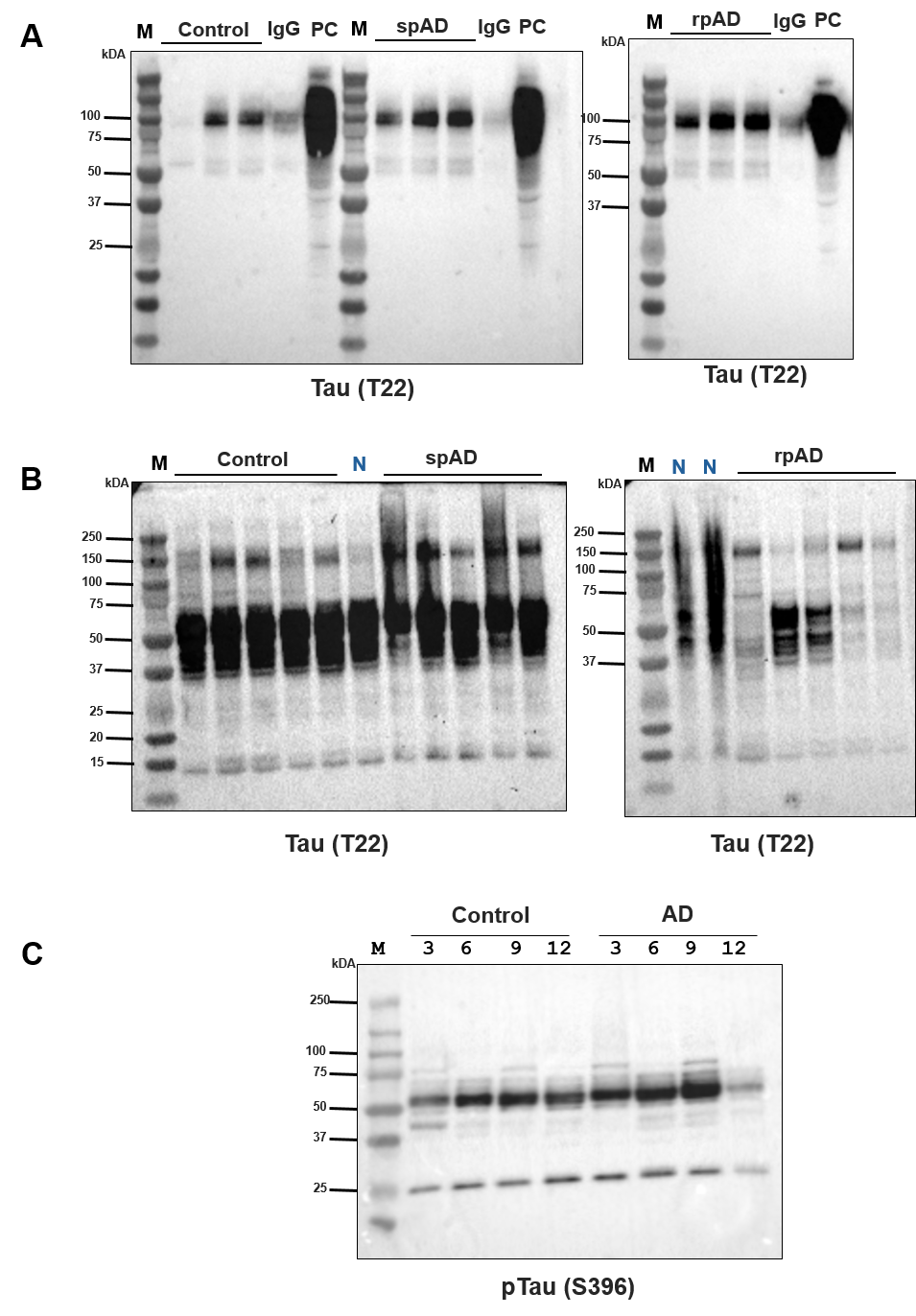


**Fig 3S. (A)** Uncropped T22 Western blot corresponding to Figure 1 in the main manuscript, showing Tau oligomer immunoprecipitation from control, spAD, and rpAD brain samples, including IgG pull-down controls. (B) Uncropped pTau (T22) Western blot corresponding to Supplementary Figure 1S, showing full-length blot. Lanes labeled “N” represent non-AD samples that were included as processing controls but were not analyzed or interpreted in the manuscript. (C) Uncropped S396 Western blot corresponding to Figure 3D in the main manuscript.

Table 1S: Summary of cases. Control; spAD: sporadic AD; rpAD: rapid Alzheimer disease; N: normal; Braak NFT: Braak neurofibrillary tangle pathology (0-VI); TAP: Thal αB phase (1-5); CERAD: Consortium to Establish a Registry for Alzheimer disease (C0-C3); NIA score: National Institute on Aging (A0-A3); PMI: postmortem delay. ABC categorization: A: TAP: amyloid score; B: Baak NFT pathology; C: CERAD (CERAD-NIA-AA score)

| **No.** | **Case** | **Clinical diagnosis** | **Age** | **Gender** | **Disease duration (M)** | **PMI hours:minutes** | **Braak NFT** | **CERAD-(NIAAA score)** | **Thal** |
| --- | --- | --- | --- | --- | --- | --- | --- | --- | --- |
| 1 | Control 1 | N | 73 | M | - | 91 | - | A0-1B0 |  |
| 2 | Control 2 | N | 62 | M | - | 48 | - | A0-1 B0 |  |
| 3 | Control 3 | N | 59 | M | - | 76 | - | A0-1 B0 |  |
| 4 | Control 4 | N | 62 | M | - | 89 | - | A0-1 B0 |  |
| 5 | Control 5 | N | 61 | M | - | 80 | - | A0-1 B0 |  |
| 6 | spAD 1 | AD; amyloid angiopathy | 81 | F | - | 05:15 | V | C |  |
| 7 | spAD 2 | AD | 82 | F | - | 01:45 | V | B |  |
| 8 | spAD 3 | AD; amyloid angiopathy ;aging related tau astrogliopathy | 75 | M | - | 09 | V | C |  |
| 9 | spAD 4 | AD ;LBD amygdala | 82 | M | - | 3:15 | V | C |  |
| 10 | spAD 5 | AD, LBD, cerebrale amyloid angiopathy | 78 | M | 6 | 24 | V | C |  |
| 11 | rpAD 1 | AD | 84 | M | 3 | 72 | V | B | 3 |
| 12 | rpAD 2 | AD | 72 | F | 2 | 144 | VI | C | 3 |
| 13 | rpAD 3 | AD, cerebrale amyloid angiopathy | 71 | F | 12 | 96 | VI | C | - |
| 14 | rpAD 4 | AD | 77 | M | 12 | 24 | VI | C | 4 |
| 15 | rpAD 5 | AD | 80 | F | 20 | 264 | V | C | - |

### ****Supplementary Table 1. Overlap and distribution of TauO-associated proteins across groups****

Summary of proteins identified in TauO co-immunoprecipitates from Control, spAD, and rpAD cases. The table includes counts of proteins unique to each group as well as proteins shared between pairwise combinations and across all three groups. Detailed protein lists for each category are provided in separate sheets within the file.

### ****Supplementary Table 2. DirectDIA quantitative proteomics dataset of TauO co-immunoprecipitates****

Comprehensive protein identification and LFQ data generated using Spectronaut (directDIA workflow). The file includes quantitative data for Control, spAD, and rpAD samples.

### ****Supplementary Table 3. Differential expression analysis of TauO-associated proteins****

Results of differential protein abundance analyses comparing:

- spAD vs Control
- rpAD vs spAD
- rpAD vs Control

For each protein, the table provides UniProt accession, gene name, protein description, log2 fold change, t-statistic, and p-value.
